# Acute Intestinal Obstruction Caused by Dual‐Component Metastasis From Combined Small Cell Lung Cancer: A Rare Case and Literature Review

**DOI:** 10.1002/ccr3.72160

**Published:** 2026-03-02

**Authors:** Wei Jiang, Xiaohui Zhou, Xizhang Zhu

**Affiliations:** ^1^ Department of Pathology Dianjiang General Hospital China; ^2^ Department of Pathology Dianjiang Hospital, Chongqing University of Chinese Medicine Chongqing China

**Keywords:** acute intestinal obstruction, case report, combined small cell lung cancer (C‐SCLC), heterogeneous metastasis, immunohistochemistry (IHC), tumor metastasis

## Abstract

This case report describes a 75‐year‐old male patient who was admitted due to acute intestinal obstruction. The patient had a history of small cell lung cancer. Emergency surgery pathology confirmed the presence of primary composite small cell lung cancer with rare independent dual component metastasis: The colonic lesion was metastatic poorly differentiated adenocarcinoma, and the mesenteric lymph nodes were metastatic small cell carcinoma. Postoperatively, the patient rapidly progressed to septic shock and multiple organ failure, ultimately resulting in death. To our knowledge, this case is the first report of Combined Small Cell Lung Cancer (C‐SCLC) presenting a rare pattern of independent dual‐component metastasis (adenocarcinoma component metastasizing to the colonic mucosa and small cell carcinoma component metastasizing to the lymph nodes) leading to acute intestinal obstruction. This finding expands the understanding of the metastatic heterogeneity of C‐SCLC, suggesting that its different cellular components may possess independent metastatic potential and target organ preferences, reflecting the molecular plasticity of tumors during evolution.

## Key Clinical Message

A 75‐year‐old male underwent surgery for intestinal obstruction, with pathology showing metastatic adenocarcinoma of the colon and small cell carcinoma of the lymph nodes, confirmed as dual‐component metastatic pulmonary composite small cell carcinoma. He rapidly deteriorated postoperatively and died 14 days after discharge.

## 1 Introduction

Combined small cell lung cancer (C‐SCLC) is a special type of malignant tumor in the lungs, referring to small cell lung cancer accompanied by non–small cell cancer (NSCC) components, including large cell neuroendocrine carcinoma (LCNEC), large cell carcinoma (LCC), adenocarcinoma, squamous cell carcinoma, spindle cell carcinoma, or giant cell carcinoma [1, 2, 3, 4]. When small cell lung cancer is accompanied by LCNEC or LCC, the proportion of LCNEC or LCC components is ≥ 10%, while there are no proportion requirements when accompanied by other NSCC components. The incidence of C‐SCLC among all small cell lung cancers is 2%–24%, and it is characterized by high malignancy, early metastasis, and an extremely poor prognosis [5, 6, 7, 8]. Although C‐SCLC is sensitive to initial chemoradiotherapy treatment, about 80% of patients experience recurrence within 6 months posttreatment, with a 5‐year survival rate of ≤ 10%. Its metastases often involve the liver, brain, and bones; however, metastases of distinct tumor components to separate anatomical sites independently—such as adenocarcinoma metastasizing to the colonic mucosa and SCLC metastasizing to lymph nodes—have not been reported [9, 10]. This may reflect the molecular heterogeneity and plasticity of the tumor. This case of C‐SCLC with dual components independently metastasizing to cause acute intestinal obstruction is the first report of such a metastatic pattern leading to intestinal obstruction, aiming to expand the understanding of C‐SCLC metastatic heterogeneity, diagnostic challenges (such as the importance of repeat biopsy and genetic testing), and clinical management [9].

## 2 Case Presentation/Examination

### 2.1 History and Presentation

The patient is a 75‐year‐old male admitted due to abdominal distension and pain accompanied by cessation of gas and bowel movements for 6 days. The abdominal pain initially presented as intermittent and tolerable but progressively worsened to persistent dull pain with paroxysmal exacerbations, without accompanying symptoms such as nausea, vomiting, or fever. After symptom onset, he was diagnosed with intestinal obstruction at a local health center; however, symptomatic treatments, including fasting and intravenous fluids, were ineffective. The patient has a history of pulmonary nodules. In May 2021, pulmonary nodules were identified, and a CT‐guided percutaneous lung biopsy was performed. The pathological diagnosis was consistent with small cell carcinoma. Immunohistochemistry results showed the following: CK (+), P40 (−), CK7 (−), TTF‐1 (+), Syn (+), CgA (+), CD56 (+), Ki‐67 (50% +), ALK (D5F3) (−), ALK‐P (+), ALK‐W (−). The patient previously received chemotherapy (specific regimen unknown; family members reported that follow‐up examinations showed tumor shrinkage). More than 8 months ago, due to acute abdominal pain, he underwent laparoscopic exploration at this hospital. Intraoperative findings included the following: normal liver size and shape with rosy color, no nodules or masses; gallbladder not enlarged, without edema or congestion; spleen normal in size and shape, rosy in color, no nodules or masses; stomach and duodenum without edema or dilation; no nodules in the mesentery and no fluid accumulation in the abdominal cavity. With the patient in the head‐down and feet‐up position, the greater momentum was pushed upward to expose the small intestine, which was examined sequentially from the ligament of Treitz to the cecum, showing no dilation, edema, congestion, ischemic necrosis, or torsion. The ascending, transverse, descending, and sigmoid colon also showed no dilation, edema, congestion, ischemic necrosis, or torsion. No enlarged lymph nodes were found in the retroperitoneum. Intraoperative exploration revealed no abnormalities in the abdominal cavity. The postoperative diagnosis considered functional abdominal pain and possible cancer‐related pain, with no clear metastatic lesions identified.

Since the onset of this illness, the patient has been experiencing low spirits, poor sleep, and a decreased appetite, alongside a persistent cessation of gas and bowel movements. However, he has maintained normal urine output and has not shown any significant changes in weight or body mass index, which is recorded at 22.6. The patient was admitted on September 6, 2022, due to abdominal pain of unknown origin. During the physical examination, his vital signs were stable, but he appeared to be in acute distress. The heart and lung examinations were unremarkable. Upon examination of the abdomen, it was found to be distended, and there were no visible gastrointestinal peristaltic waves. The lower and left abdomen displayed well‐healed laparoscopic puncture incisions. Tenderness was noted throughout the abdomen, with the most significant discomfort located in the lower middle region.

### 2.2 Routine Laboratory

The laboratory tests (at admission) results are shown in Table 1: Laboratory investigations at admission revealed several abnormalities. Inflammatory markers were notable for an elevated high‐sensitivity C‐reactive protein (hsCRP) level of 56.97 mg/L and an increased monocyte percentage of 18.7%. The complete blood count showed mild anemia with a hemoglobin level of 115 g/L. Liver function tests indicated cholestasis with elevations in alkaline phosphatase (ALP, 182 U/L) and γ‐glutamyl transferase (GGT, 242 U/L). Renal function and electrolyte panels demonstrated mild renal impairment (creatinine 114.5 μmol/L), hypocalcemia (2.02 mmol/L), and mild hypokalemia (3.57 mmol/L). Coagulation studies were significant for a markedly elevated D‐dimer level of 11.98 mg/L, suggesting a hypercoagulable state. Tumor marker assays showed significant elevations, particularly in carbohydrate antigen 15–3 (CA15‐3, 564.6 U/mL), neuron‐specific enolase (NSE, 38.12 ng/mL), carbohydrate antigen 125 (CA125, 62.6 U/mL), and cytokeratin fragment 21–1 (CYFRA21‐1, 8.46 ng/mL); carcinoembryonic antigen (CEA) was mildly elevated at 7.09 ng/mL.

**TABLE 1:** Laboratory tests.

Test item	Result	Unit	Reference range
Hb	115 ↓	g/L	130–175
hsCRP	56.97 ↑	mg/L	< 5
ALP	182 ↑	U/L	45–125
GGT	242 ↑	U/L	10–60
Cr	114.5 ↑	μmol/L	59–104
D‐Dimer	11.98 ↑	mg/L	< 0.5
CEA	7.09 ↑	ng/ml	< 5
CA125	62.6 ↑	U/ml	< 35
CA15‐3	564.6 ↑	U/ml	< 25
CYFRA21‐1	8.46 ↑	ng/mL	< 3.3
NSE	38.12 ↑	ng/mL	< 16.3

Abbreviations: ALP, alkaline phosphatase; CA125, carbohydrate antigen 125; CA15‐3, carbohydrate antigen 15–3; CEA, carcinoembryonic antigen; Cr, creatinine; CYFRA21‐1, cytokeratin 19 fragment; GGT, gamma‐glutamyl transferase; Hb, hemoglobin; hsCRP, high‐sensitivity C‐reactive protein; NSE, neuron‐specific enolase; WBC, white blood cell count.

### 2.3 Imaging Findings

**FIGURE 1:**
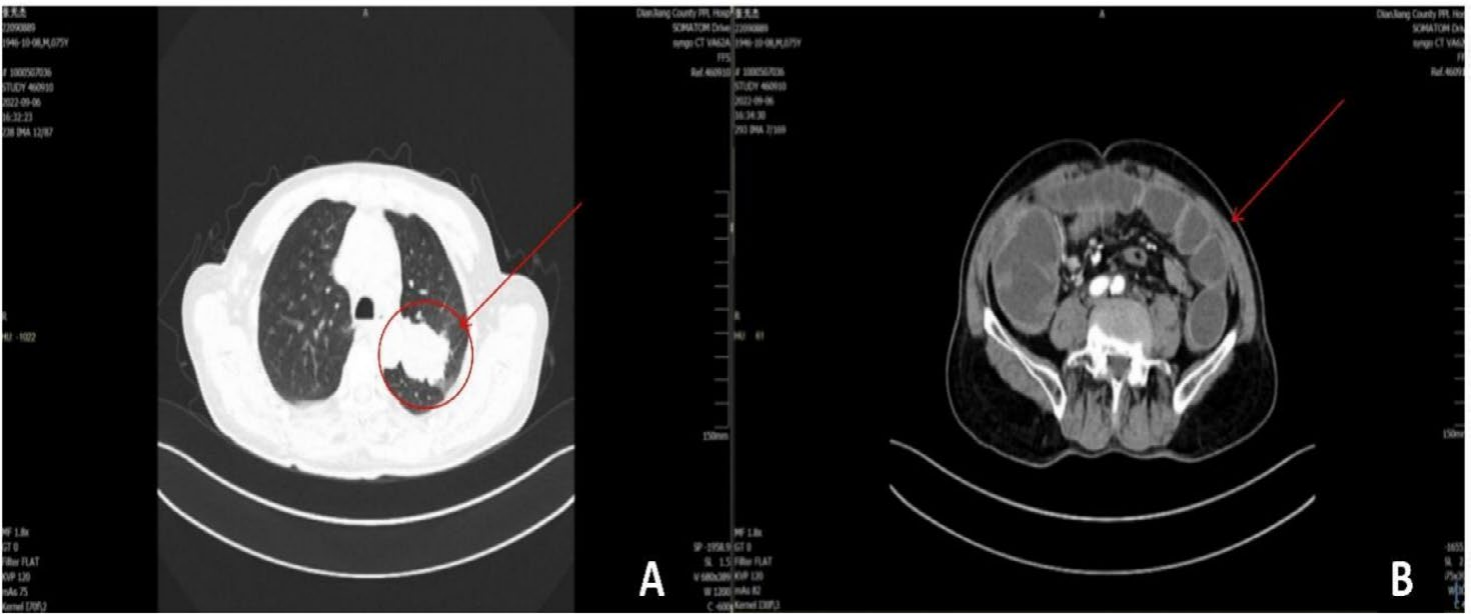
Imaging examination: Contrast‐enhanced chest CT shows a mass in the left upper lobe (5.8 cm × 4.2 cm), suggesting progression of the patient's primary lung cancer (Figure 1A). Contrast‐enhanced abdominal CT shows intestinal volvulus with a “vortex sign,” supporting the radiological diagnosis of intestinal obstruction (Figure 1B).

Electrocardiogram examination: T wave mildly abnormal. The CT Scan results are shown in Figure 1: Figure 1A shows the mass in the posterior segment of the left upper lobe is significantly increased compared with the film on March 3, 2022 (2.0 cm * 1.2 cm → 5.8 cm * 4.2 cm). There is a small amount of new pleural effusion on both sides and partial lung consolidation in the right lower lung. Other findings are essentially unchanged compared with those in previous imaging. Figure 1B exhibits CT scan showing intestinal obstruction and spiral‐shaped blood vessels in the mid‐abdomen, suggesting possible intestinal torsion. A small amount of fluid is present in the paracolic gutters on both sides.

Enhanced CT (Figure 1A,B):

### 2.4 Surgery and Intraoperative Findings

A patient presented with progressively worsening abdominal pain, and imaging studies indicated a mechanical obstruction, suspected to be an intestinal volvulus. Conservative treatment failed to alleviate the symptoms, prompting the surgical team to proceed with an emergency exploratory laparotomy after ruling out any absolute contraindications for surgery. During the operation, the findings revealed that the proximal small intestine was congested and edematous, although it did not show significant dilation. In contrast, the distal small intestine was notably dilated, measuring approximately 4 cm in diameter, while the ascending colon was significantly enlarged, with a diameter of about 12 cm. The intestinal wall appeared thin and was under extreme tension. Additionally, a palpable mass was detected in the hepatic flexure of the colon, measuring around 4 cm × 3 cm × 3 cm; it was hard in texture, poorly mobile, and surrounded by stiff tissues with indistinct boundaries. Given these intraoperative observations, the surgical team performed several procedures, including exploratory laparotomy, intestinal decompression, right hemicolectomy, and ileostomy.

### 2.5 Pathological Examination

Gross examination: The resected portions include the right colon, ileum, cecum, appendix, and part of the colon. The ileum is 10 cm long and 4 cm in diameter; the colon is 20 cm long and 5–7 cm in diameter; and the appendix is 5 cm long and 0.5 cm in diameter. Upon dissection of the specimen, a grayish‐white mass measuring 4.0 cm × 1.0 cm × 0.5 cm is observed in the submucosa, located 7 cm from the proximal margin of the colon. The mass invades the muscularis propria and has a grayish‐white cut surface with a medium texture. The cecum is dilated, and the intestinal wall is thin, measuring 0.1–0.2 cm in thickness; it appears dark brown, with a perforation 0.5 cm in diameter visible. Fifteen lymph nodes, measuring 0.3–1.5 cm in diameter, are found within the adipose tissue on the surface of the intestinal wall.

Pathological diagnosis of right hemicolon revealed metastatic poorly differentiated adenocarcinoma of the colon involving the submucosa and muscularis, and no residual tumor tissue was seen at the margins of excision. Metastatic small cell carcinoma was found in 4 of 15 mesenteric lymph nodes.

The immunohistochemistry (IHC) results are shown in Table 2.

**TABLE 2:** Comparison of immunohistochemistry results.

Biomarker	Colonic lesion	Mesenteric lymphadenopathy	Meaning
TTF‐1	2+	2+	Both metastatic foci are of pulmonary origin
CK7	3+	—	Characteristics of adenocarcinoma
CD56	—	2+	Neuroendocrine differentiation
Syn	—	2+	Neuroendocrine differentiation
CgA	—	+	Neuroendocrine differentiation
Napsin A	2+	—	Characteristics of lung adenocarcinoma
CDX2	—	—	Exclude primary gastrointestinal cancer
Villin	—	/	Exclude primary gastrointestinal cancer
CEA	—	/	Exclude primary gastrointestinal cancer
Ki‐67	3 + 10%	3 + 40%	The proliferation activity of SCLC components is significantly higher
LCA	—	/	Excluding lymphoma
P40	—	/	Exclude lung squamous cell carcinoma
CK5/6	—	/	Exclude lung squamous cell carcinoma
S‐100	—	/	Exclude neurogenic tumors

2206632‐3 (colon) IHC: CK7 (3+), TTF‐1 (2+), Napsin A (2+), p63 (−), CK5/6 (−), p40 (−), Ki‐67 (3+, 10%), CD56 (−), Syn (−), CK (3+), Cg A (−), NSE (−), S‐100 (−), LCA (−), CDX2 (−), Villin (−), CEA (−).

2206632‐8 (Mesenteric lymph nodes) IHC: CD56 (2+), Syn (2+), TTF‐1 (2+), CgA (+), NSE (3+), Ki‐67 (3+, 40%).

#### 2.5.1 Pathological interpretation (Figures 2, 3, 4, 5)

**FIGURE 2:**
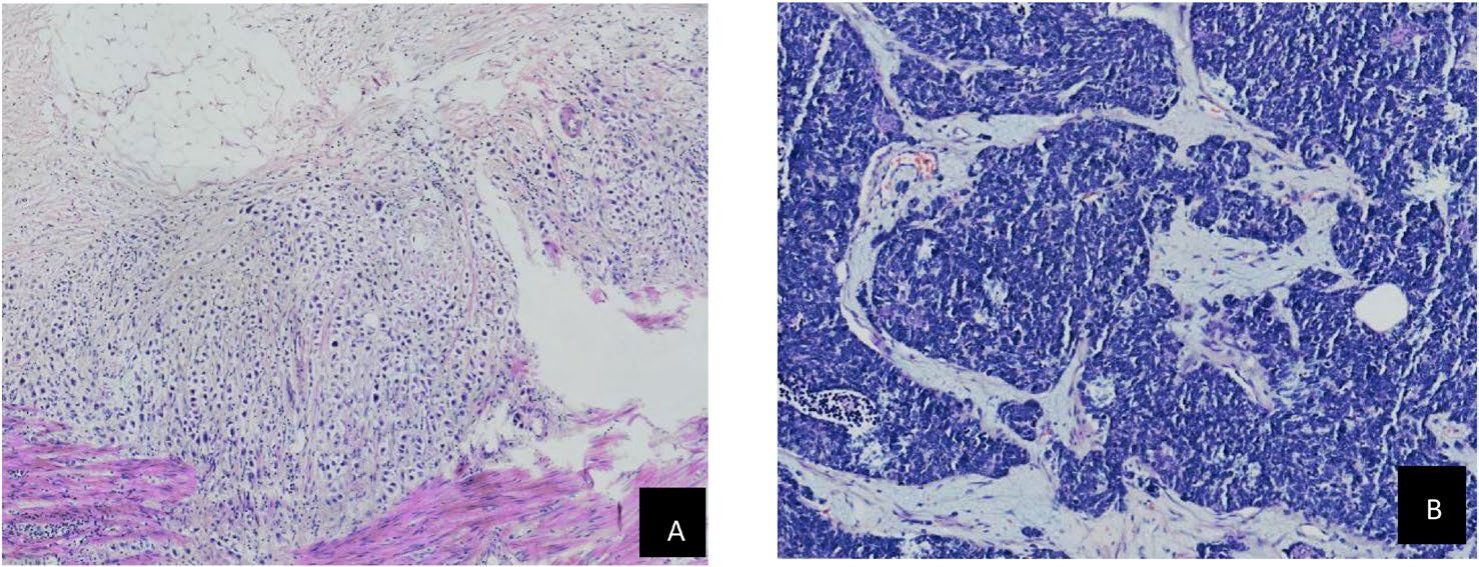
(A) Pathological examination results of colon tumor HE (hematoxylin and Eosin) × 400. (B) Pathological examination results of Mesenteric lymph nodes HE × 400.

**FIGURE 3:**
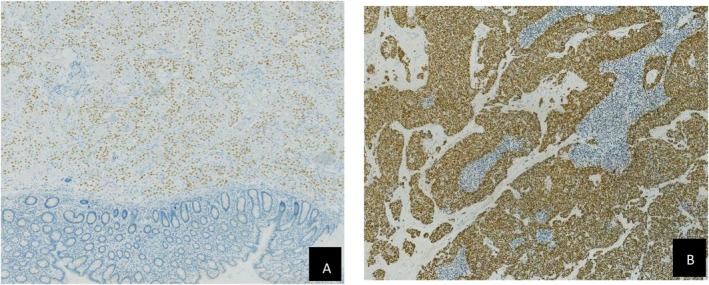
(A) Pathological examination results of colon tumor TTF‐1 (+) IHC × 400. (B) Pathological examination results of mesenteric lymph nodesTTF‐1 (+) IHC × 40.

**FIGURE 4:**
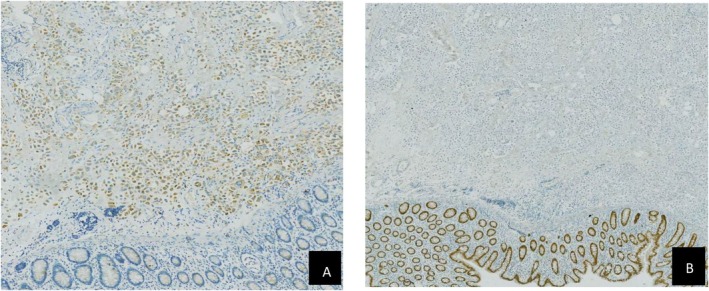
(A) Pathological examination results of colon tumor Napsin A (+) IHC × 400. (B) Pathological examination results of colon tumorCDX2 (−) IHC × 400.

**FIGURE 5:**
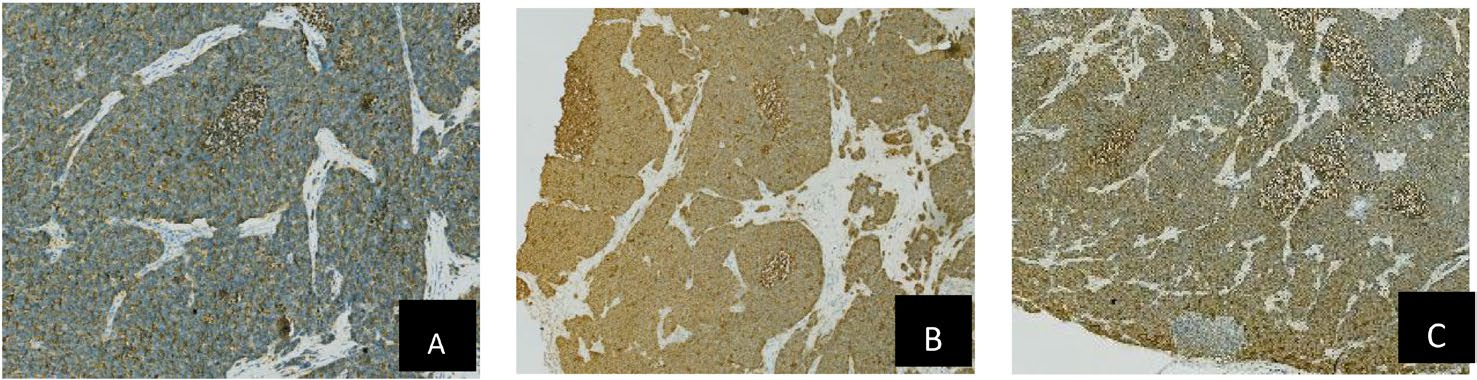
(A) pathological examination results of mesenteric lymph nodes Syn (+) IHC × 400. (B) Pathological examination results of mesenteric lymph nodes CD56 (+) IHC × 400. (C) Pathological examination results of mesenteric lymph nodes NSE (+) IHC × 40.

### 2.6 Conclusion and Results

The core diagnostic issue in this case is to clarify the cause of acute abdomen and intestinal obstruction, specifically the nature of the colonic neoplasm (primary or metastatic) and its relationship with the patient's history of small cell lung cancer (SCLC). Through systematic, pathological, and immunohistochemical analysis, the diagnosis and differential diagnosis process are as follows.

#### 2.6.1 Differentiation of the Nature of the Colonic Neoplasm

The colonic neoplasm discovered due to intestinal obstruction needs to be first differentiated as primary colon cancer or metastatic cancer. Given the patient's history of SCLC, metastatic tumors need to be considered primarily. However, there are significant differences in histomorphology between the intestinal tumor and the mesenteric lymph node metastases, suggesting possible different origins or heterogeneous manifestations of the same primary tumor. To clarify the diagnosis, systematic immunohistochemical examinations were conducted: Exclusion of lung adenocarcinoma origin: TTF‐1 (thyroid transcription factor‐1) and Napsin A were tested, both of which are relatively specific markers for lung adenocarcinoma, and negative results can exclude colonic metastasis from lung adenocarcinoma. Exclusion of lung squamous carcinoma origin: p63, CK5/6, P40, and other squamous carcinoma markers were tested, and negative results can exclude lung squamous carcinoma metastasis. For the exclusion of neuroendocrine tumors, CD56, Syn (synaptophysin), and CgA (chromogranin A) were tested. These are broad‐spectrum markers for neuroendocrine tumors, which are usually positive in SCLC. The detection of these markers in the colonic neoplasm aims to differentiate between neuroendocrine tumors originating in the colon or metastasis from SCLC. Negative results can exclude neuroendocrine tumors. For the exclusion of gastrointestinal primary adenocarcinoma, CDX2 (caudal‐type homeobox transcription factor 2), Villin (Villin), and CEA (carcinoembryonic antigen) were tested. These are commonly used markers for gastrointestinal adenocarcinoma, and positive results support gastrointestinal origin. If the immunophenotype of the colonic neoplasm does not match, it supports its metastatic nature.

#### 2.6.2 Confirmation of Mesenteric Lymph Node Metastases

Cancer metastasis was found in the mesenteric lymph nodes, and its histomorphology was assessed to be consistent with characteristics of small cell carcinoma. Morphology reveals Tumor cells are small in size, chromatin is finely granular, nucleoli are not prominent, and cytoplasm is scant. Immunohistochemistry should express neuroendocrine markers (such as CD56, Syn, CgA) and epithelial markers (such as CK). Although some squamous carcinoma markers (such as P40, P63) may be focally positive in small cell lung cancer, the combination of morphology and positive neuroendocrine markers can confirm the diagnosis. This lymph node metastasis was confirmed by immunohistochemistry to be metastatic small cell carcinoma, consistent with the patient's previous SCLC history, supporting it as a metastasis from primary lung SCLC.

### 2.7 Postoperative Course and Outcome

The patient's condition progressively deteriorated postoperatively. Within 24 h after surgery, he progressed to septic shock and multiple organ failure, manifesting as ventilator dependency, acute kidney injury requiring renal replacement therapy, and coagulopathy. His postoperative course was characterized by continued clinical decline. The family opted for discharge home on postoperative Day 14, and subsequent follow‐up confirmed his death.

## 3 Discussion

This case represents the first reported instance of combined small cell lung cancer manifesting independent metastasis of its adenocarcinoma and small cell carcinoma components to distinct anatomical sites (colonic mucosa and mesenteric lymph nodes, respectively), culminating in acute mechanical bowel obstruction [11, 12]. While C‐SCLC is known for aggressive metastasis, prior reports primarily describe dissemination of the tumor as a whole or with mixed components to common sites like the liver, brain, or bones. The phenomenon of divergent histological components “seeding” separate organ systems independently is exceedingly rare, and such a pattern leading to acute intestinal obstruction has not been documented previously [12, 13, 14, 15].

This unique metastatic behavior underscores the profound intratumoral heterogeneity and molecular plasticity of C‐SCLC. It suggests the coexistence of subclones within the primary tumor with distinct metastatic propensities. The “seed and soil” hypothesis offers a plausible framework: Adenocarcinoma cells (the “seed”), likely via hematogenous spread, may find a conducive niche in the vascular‐rich submucosa of the colon (the “soil”) [16, 17], whereas a subpopulation of small cell carcinoma cells with potent lymphotrophic affinity may preferentially colonize regional lymph nodes via lymphatic channels. The shared TTF‐1 positivity in both metastatic foci confirms a common pulmonary origin, while the divergent metastatic routes and target organs reflect underlying differences in driver mutations, epigenetic programming, or microenvironmental interactions, representing an adaptive outcome of clonal selection during tumor evolution.

This case highlights significant diagnostic challenges. In lung cancer patients (particularly with SCLC/C‐SCLC) presenting with acute bowel obstruction, differential diagnoses must include the following: (1) the rare mechanical obstruction caused by metastatic deposits, as seen here; and (2) paraneoplastic chronic intestinal pseudo‐obstruction, an autoimmune disorder mediated by anti‐Hu/Yo antibodies damaging the myenteric plexus, which presents without a mechanical obstructing point on imaging and is managed medically, with surgery being ineffective or detrimental [18, 19, 20]. CT imaging is the cornerstone for differentiation. Pathological confirmation is paramount. When metastasis is suspected, systematic immunohistochemical staining (e.g., TTF‐1, Napsin A, CDX2, synaptophysin, chromogranin A) is essential to delineate components and origin. Notably, approximately 30% of lung adenocarcinoma gastrointestinal metastases can be CDX2‐negative, underscoring the need for integrating clinical history with a panel of markers including TTF‐1 and CK7.

Therapeutically, this case reveals a profound dilemma and offers critical insights. Although emergency surgery, aligned with principles for managing oligometastatic complications, successfully relieved the obstruction and provided pathological diagnosis, the patient's rapid systemic deterioration postoperatively underscores the limited efficacy of surgery against the inherent aggressiveness and potential occult micrometastases of C‐SCLC. This emphasizes the critical importance of perioperative multidisciplinary team assessment [21]. Decision‐making must balance the life‐saving benefit of emergency intervention against the risk of accelerated systemic progression. The central tenet is that surgery must not unduly delay the initiation of systemic therapy. For C‐SCLC, treatment strategies should address both the SCLC component (platinum‐etoposide chemotherapy) and the NSCLC component (considering targeted or immunotherapy based on biomarkers) [22, 23]. Therefore, a rapid preoperative systemic evaluation should be attempted, and systemic therapy should be initiated or adjusted at the earliest feasible opportunity postoperatively, rather than relying solely on local intervention.

In conclusion, this case expands the understanding of metastatic heterogeneity in C‐SCLC. The pattern of dual‐component independent metastasis carries important educational and cautionary implications [24]. It calls for heightened clinical vigilance toward the complex behavior of such tumors, advocates for an integrated diagnostic approach combining imaging and pathology, and mandates a management strategy rooted in multidisciplinary collaboration with early systemic therapy at its core, aiming to optimize care for patients facing this formidable disease [25, 26, 27].

## 4 Conclusion

This case highlights the rare occurrence of synchronous independent metastases from both adenocarcinoma and small cell carcinoma components in combined small cell lung cancer (C‐SCLC), reflecting its aggressive biology and clonal divergence [28]. Despite radical resection of the oligometastases, rapid progression ensued, underscoring the high risk of occult micrometastases and the inadequacy of localized therapy alone. Our findings convey crucial clinical implications: Comprehensive histopathological evaluation via multisite biopsies is essential to overcome sampling bias [29, 30], and early initiation of systemic therapy within a multidisciplinary framework is paramount—genetic testing for targeted therapy should be considered if non‐squamous NSCLC components are present, while an immune‐checkpoint inhibitor combined with chemotherapy may be an option for squamous components [31]. Future efforts should employ multi‐omics profiling to decipher the molecular heterogeneity of C‐SCLC, which will be critical to guide the development of precision oncology strategies and improve outcomes for these patients with poor prognosis [32].

## Author Contributions

**Xiaohui Zhou:** conceptualization, data curation, methodology, supervision, visualization, writing – original draft. **Wei Jiang:** conceptualization, data curation, investigation, visualization, writing – original draft. **Xizhang Zhu:** conceptualization, project administration, supervision, writing – review and editing.

## Funding

The authors have nothing to report.

## Ethics Statement

The Ethics Committee of the Dianjiang General Hospital, Chongqing (DYLL‐KY‐2025‐20), provided approval for the study.

## Consent

Written informed consent was obtained from the individual for the publication of any potentially identifiable images or data included in this article.

## Conflicts of Interest

The authors declare no conflicts of interest.

## Data Availability

Data sharing not applicable to this article as no datasets were generated or analysed during the current study.
